# Orchestrating Asymmetric Expression: Mechanisms behind *Xist* Regulation

**DOI:** 10.3390/epigenomes8010006

**Published:** 2024-02-01

**Authors:** Samuel Jesus Luchsinger-Morcelle, Joost Gribnau, Hegias Mira-Bontenbal

**Affiliations:** Department of Developmental Biology, Erasmus MC, University Medical Center, 3015 GD Rotterdam, The Netherlands; s.luchsingermorcelle@erasmusmc.nl (S.J.L.-M.); j.gribnau@erasmusmc.nl (J.G.)

**Keywords:** X chromosome inactivation, *Xist*, epigenetics, lncRNAs, asymmetric expression

## Abstract

Compensation for the gene dosage disequilibrium between sex chromosomes in mammals is achieved in female cells by repressing one of its X chromosomes through a process called X chromosome inactivation (XCI), exemplifying the control of gene expression by epigenetic mechanisms. A critical player in this mechanism is *Xist*, a long, non-coding RNA upregulated from a single X chromosome during early embryonic development in female cells. Over the past few decades, many factors involved at different levels in the regulation of *Xist* have been discovered. In this review, we hierarchically describe and analyze the different layers of *Xist* regulation operating concurrently and intricately interacting with each other to achieve asymmetric and monoallelic upregulation of *Xist* in murine female cells. We categorize these into five different classes: DNA elements, transcription factors, other regulatory proteins, long non-coding RNAs, and the chromatin and topological landscape surrounding *Xist*.

## 1. Introduction

Dosage compensation of gene expression between chromosomes is essential for survival. Female mammalian cells carry two X chromosomes, while male cells carry an X and a Y chromosome, generating an X-linked gene dosage imbalance between the sexes. To achieve dosage compensation, female diploid epiblast cells inactivate a single X chromosome very early during embryonic development via a complex process known as X chromosome inactivation (XCI). This process results in the epigenetic silencing of one randomly selected X chromosome (Xi), indicating that female and male cells must sense how many X chromosomes they carry.

In mice, two waves of epigenetic silencing occur. Firstly, imprinted XCI (iXCI) leads to the inactivation of the paternal X chromosome following fertilization. This paternal Xi is later re-activated in the inner cell mass of the embryo, and a second, random XCI (rXCI) wave occurs, where a single X is randomly chosen for inactivation in the early epiblast. Most in vitro studies of XCI are performed in mouse embryonic stem cells (ESCs), which carry two active X chromosomes (Xa) and initiate rXCI upon exit of pluripotency and differentiation, recapitulating events in the epiblast of embryos. However, not all genes on the Xi are silenced; they are called escapees or escaping genes. Even though XCI happens in all mammals, different species show different patterns of it. While mice display both types of XCI, rabbits, monkeys, and humans, for instance, only show rXCI, and marsupials display iXCI only. This illustrates the variety of XCI mechanisms present within the mammalian class. New data are emerging on different mechanisms in other mammals, such as rabbits, monkeys and especially humans (reviewed in [1]). However, since most XCI research has been performed in mice and very exciting new data are still being generated nowadays, we focus this review on mouse rXCI.

### 1.1. Kicking off X Chromosome Inactivation: The Stochastic Model

In 1971, Mary Lyon proposed a model where a diffusible X-linked factor would guarantee XCI of a single X [2]. This model has been further developed into a stochastic model, where female cells sense X chromosome dosage, leading to the inactivation of a single X chromosome while preventing inactivation of the single X chromosome in male cells. The stochastic model proposes that XCI is achieved by a tightly controlled balance between X-encoded activators and autosomally encoded repressors [3]. Many autosomally encoded repressors are pluripotency factors or are linked to the pluripotent state and therefore prevent the inactivation of an X chromosome in the inner cell mass of the embryo (ICM) or in ESCs. ESCs are derived from the ICM, which will give rise to the embryo proper. Upon differentiation, the downregulation of repressors of XCI and upregulation of activators of XCI would tilt the balance towards XCI. Thus, female exclusive activation of XCI is mediated by the double dose of X-encoded XCI activators that are required to overcome the threshold set by the XCI repressors. Initiation of XCI is stochastic, and a negative feedback loop involving rapid silencing of some XCI activators prevents inactivation of the second X chromosome. The application of novel machine learning and mathematical modeling techniques indicates that XCI dynamics can be recapitulated based on the principles of the stochastic model with a limited number of activators and repressors regulating XCI [4,5].

### 1.2. The X Inactivation Center and the Tsix/Xist Tandem

**Figure 1:**
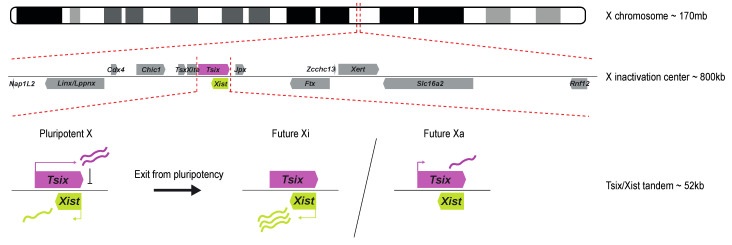
Schematic overview of the X inactivation center and the *Tsix*/*Xist* tandem. The Xic is located at ~103 Mb on the mouse X chromosome (mm10). It contains several lncRNAs, such as *Linx*/*Lppnx*, *Tsx*, *Xite*, *Tsix*, *Xist*, *Jpx*, *Ftx*, and *Xert*, and several coding genes, such as *Nap1L2*, *Cdx4*, *Chic1*, *Slc16a2*, and *Rnf12*. *Xist* and *Tsix* are two antisense genes at the center of the Xic. In the pluripotent state, their expression is symmetric between both X chromosomes: *Tsix* is biallelically expressed and represses *Xist*, leading to low levels of *Xist* expression. Upon XCI, *Tsix* is downregulated from the future Xi, resulting in monoallelic *Xist* upregulation, while the future Xa maintains low levels of *Tsix*, further suppressing *Xist* expression. The symmetry is broken, and XCI happens on a single chromosome in diploid female cells.

Research on truncated human X chromosomes and balanced X-autosome translocations led to the discovery of the X inactivation center (Xic), a locus on the X chromosome essential for XCI [6,7]. The mouse Xic spans many long non-coding RNAs and several protein-coding genes, covering at least 800kb (reviewed in [8]). Later, transgenic studies showed the long non-coding RNA (lncRNA) gene *Xist*, embedded within the Xic, to be required for XCI to happen. *Xist* is critical for both iXCI and rXCI in mice. Endogenous *Xist* deletions prevent XCI from happening on the X chromosome carrying the deletion, while ectopic insertions on autosomes result in their silencing [9,10]. The mouse *Xist* gene is a 22 kb-long gene with no conserved open reading frame (ORF) and is transcribed, polyadenylated, and alternatively spliced into a 15 kb-long lncRNA [11,12,13]. *Xist* is transcribed antisense to another lncRNA named *Tsix* that spans *Xist* entirely and negatively regulates its expression during development [14]. Biallelic expression of *Tsix* in the pluripotent state thus maintains the silent state of *Xist* in female ESCs. Exit of pluripotency triggers a break from symmetric *Xist* expression to an asymmetric *Tsix*/*Xist* expression state, where *Tsix* expression is maintained on the Xa while *Xist* expression is greatly upregulated on the selected Xi. Finally, *Xist* expression is locked on the Xi, and the eventual downregulation of *Tsix* from the Xa happens once XCI is established (Figure 1). Many of these types of antisense gene expression regulation have been described in animals and plants, such as imprinted genes or the Flc-COOLAIR gene-tandem involved in plant flowering [15].

In this review, we hierarchically discuss the several levels of *Xist* regulation that play a role in both the maintenance and breakage of Xic symmetry in mouse ESCs during rXCI. We have categorized the levels that contribute to the *Tsix*/*Xist* tandem expression pattern into five different classes: DNA elements, transcription factors, other proteins, lncRNAs, and the chromatin and topological landscape surrounding *Xist*.

## 2. DNA Elements That Impact *Xist* Expression

### 2.1. The Promoter Region of Xist

The discovery of the *Xist* gene was a significant breakthrough in understanding the mechanism of XCI. Its lncRNA product is now accepted as the master regulator behind the epigenetic silencing of one of the two X chromosomes in female mammals. *Xist* was first identified and isolated from a mouse cDNA library in 1991 [7]. It was discovered that *Xist* RNA spreads in *cis*, interacting with the chromatin of the X chromosome, leading to the spread of heterochromatinization and subsequent inactivation of the chromosome [12,16]. In an effort to understand the transcriptional regulation of *Xist* and thus the mechanisms kicking off XCI, follow-up studies centered on the characterization of the *Xist* promoter.

**Figure 2:**
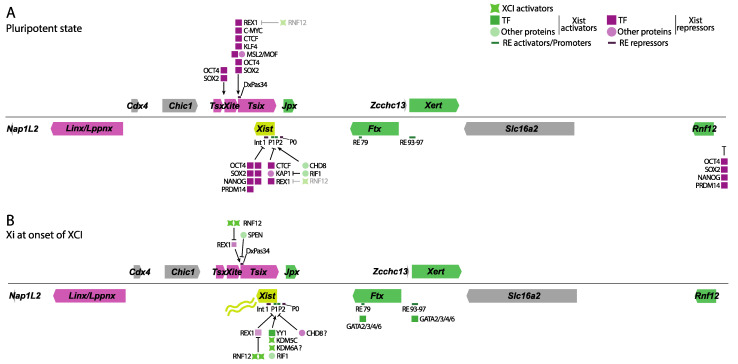
Overview of the different proteins and REs involved in *Xist* and *Tsix* regulation. Genes that have an activating direct or indirect role on *Xist* are shown in green, while genes involved in *Xist* repression are shown in magenta. Several TF, other proteins, XCI activators, and RE are shown in green or magenta based on their effect on *Xist* expression. (**A**) In pluripotency, the pluripotency factor network (OCT4, NANOG, SOX2, REX1, PRDM14) keeps *Tsix* active and represses *Xist*, either directly by binding to Intron 1 or its promoter, or indirectly through *Tsix* or by inhibiting the *Xist* activator RNF12. CTCF and KAP1 also work towards inhibiting its expression, while the MSL complex supports *Tsix* expression from its promoter. Another set of activating proteins, such as CHD8 and RIF1, supports low levels of *Xist* expression. (**B**) At the onset of XCI, reduced pluripotency factor concentrations lead to decreased *Tsix* expression. Reduction in REX1 is further aided by increased RNF12 expression due to the disappearance of its pluripotent repressors. SPEN is required to shut down *Tsix* expression to allow *Xist* upregulation. YY1, the paralog of REX1, binds to the P2 promoter of *Xist*, activating it. KDM5C and KDM6A also seem to bind there, leading to the demethylation of H3K4me2/3 to H3K4me1, a mark of enhancers, while seemingly removing H3K27me3. CHD8, however, seems to have an opposing role at the onset of XCI during differentiation because it decreases YY1s accessibility to *Xist*’s promoter. CHD8 seems to fine-tune *Xist* expression depending on the developmental context. Several of the GATA binding factors are required for *Xist* expression during differentiation by binding several of its regulatory sequences. Shades of magenta show *Xist* repressors, while shades of green indicate *Xist* activators and XCI activators.

Early studies identified the mouse *Xist* gene’s minimal promoter region to be approximately 0.4 kb in size and located right upstream of the major transcriptional start site (TSS). This minimal promoter is a weak constitutive TATA-like promoter with a possible initiator element spanning the TSS [17]. *Xist* promoter sequences have been compared between several mammals, including humans, mice, rabbits, and horses, showing a high degree of conservation between these species [18]. Follow-up studies identified multiple *Xist* promoters, termed P1, P2, and P0, with P1 being the first identified region just upstream of *Xist’s* TSS. The P0 promoter of *Xist* was mapped 6.5 kb upstream of the TSS and gives rise to unstable *Xist* transcripts. The P2 promoter was mapped 1.5 kb downstream of the TSS and thus within *Xist* exon 1, giving rise to a stable *Xist* transcript (Figure 2A,B). Promoter switching from P0 to P1/P2 was then proposed as a regulatory mechanism controlling *Xist* expression with the exit of pluripotency [19]. However, recent chromatin immunoprecipitation assays followed by sequencing (ChIP-seq) of the P2 promoter of *Xist* seem to indicate this region might act as an internal regulatory element, or enhancer, rather than a promoter [20,21].

### 2.2. Distant Regulatory Regions

While the promoter regions of *Xist* drive its expression, distant DNA regulatory elements (RE) seem to play a role in titrating its expression. A regulatory region located ∼10 kb downstream of *Xist* TSS, and thus within *Xist* intron 1, was shown to bind several pluripotency factors (see Section 3.2) and play a role in *Xist* repression in the pluripotent state (Figure 2A) [22]. A subsequent study confirmed these findings through genetic deletions of *Xist* intron 1. Transgenic male ESCs carrying an *Xist* intron 1 deletion show moderately upregulated *Xist* expression in the pluripotent state, which is exacerbated upon *Tsix* co-removal [23]. However, in two contrasting studies, deletion of *Xist* intron 1 in female ES cells does not seem to impact *Xist* expression both in vivo and in vitro but rather skews the future Xi choice towards the mutated allele [24,25].

Identification of REs involved in *Xist* expression has proved to be a challenging task due to numerous factors influencing *Xist* expression within the Xic. Distinguishing direct from indirect effects on *Xist* expression via genetic perturbations within the Xic is difficult. However, a recent publication has successfully addressed this challenge. Using an elegant screening approach taking advantage of dCas9 fused to the repressor KRAB (CRISPRi) and detecting *Xist* levels by fluorescently activated cell sorting, several candidate regions were epigenetically and systematically silenced [26]. As expected, epigenetic inactivation of the previously discussed *Xist* promoter regions, as well as of known *Xist* regulators (discussed later in this review), impairs *Xist* upregulation upon exit of pluripotency, serving as a validation of the screening approach. A novel regulatory cluster located ~150 kb upstream of the *Xist* TSS, termed RE93-97, was also identified. Targeting this cluster with CRISPRi revealed a gradual decrease in *Xist* expression, pointing to RE93-97’s role as a *Xist* enhancer cluster. Furthermore, capture Hi-C analysis revealed RE93-97 interacts with *Xist’s* P2 promoter upon exit of pluripotency, suggesting this regulatory cluster responds to differentiation cues (see Section 3.3).

In a follow-up study by the same group, another pivotal RE responsible for driving *Xist* expression was identified [27]. Employing a CRISPR activation screening approach, several protein factors that result in *Xist* expression upon their overexpression were identified. A comprehensive analysis of potential candidates and their binding sites revealed that the GATA family of transcription factors (see Section 3.3) binds not only RE93-97 but also a newly discovered regulatory element located ~100 kb upstream of the *Xist* TSS termed RE79. Genetic deletions of RE97, as well as RE79, validated previous discoveries regarding the significance of RE93-97 in rXCI while elucidating the role of RE79 in driving *Xist* upregulation expression in iXCI.

Gene transcription regulation is achieved not only by many different protein factors binding to promoters and REs, such as RNA Pol II, transcription factors (TFs), chromatin remodellers, etc., but also through complex chromatin architecture. In this next section, we will analyze which transcription factors are important for *Xist* regulation.

## 3. Transcription Factors That Impact *Xist* Upregulation

Transcription factors regulating *Xist* expression can either be autosomally or X-encoded. In this review, we call TFs or other proteins that are autosomally encoded and regulate *Tsix* or *Xist* expression as *Xist* activators or repressors, while proteins that are X-encoded and ultimately impact *Xist* expression as XCI activators. There are currently no known X-linked *Xist* repressors.

### 3.1. X-Linked TFs: XCI Activators

A limited number of X-linked TFs are involved in *Xist* regulation. Recent data have shown that X-linked TF GATA1, a widely expressed TF in vertebrates, acts as an XCI activator [27]. Using a pooled CRISPR library to upregulate target genes in a *Xist*-sensitized male ESC line, the authors were able to discover GATA1, 4, and 6 as potential activators of *Xist*. GATA1 is an X-linked gene whose overexpression leads to increased *Xist* expression in ESCs, probably mediated by its indirect effect on GATA6’s overexpression, which binds the distal *Xist* enhancer elements RE79 and RE93-97 (see Section 2.2). Although GATA factors have been shown to induce differentiation in ESCs [28], GATA1 overexpression does not lead to an obvious differentiation phenotype. Moreover, its expression is very low in ESCs, which could explain why its knockdown (KD) does not influence rXCI kinetics. As the authors pointed out, however, its role in *Xist* overexpression might happen during iXCI, when its expression peaks during preimplantation development. Indeed, triple knockout (KO) of GATA1, 4, and 6 abrogates *Xist* expression at the 8-cell stage.

### 3.2. Autosomally Encoded TFs: Xist Repressors

Several TFs have been described in the last few decades as playing a role in *Xist* repression. They are all autosomally encoded, and the majority of them belong to the pluripotency factor network. Female mouse ESCs are powerful model systems to study rXCI, harboring two Xas and initiating rXCI upon exit of pluripotency and differentiation. Many studies have delved into how the pluripotency factors OCT4, NANOG, SOX2, and REX1 mechanistically repress *Xist* in ESCs. ChIP experiments revealed that OCT4, NANOG, and SOX2 directly bind to *Xist* intron 1 (Figure 2A) [22]. Removal of OCT4 in male ESCs, which normally should not upregulate *Xist* upon differentiation, leads to a loss of NANOG and SOX2 binding to intron 1 and a concomitant increase in *Xist* expression, indicating that these factors act as repressors of *Xist* in the pluripotent state. This elegant model specifies that ESCs, in their pluripotent state, show very little *Xist* expression, while upon differentiation, downregulation of the pluripotency factor network results in the derepression and release of *Xist*. Confirming these results, as indicated above (see Section 2.2), removal of intron 1 in male ESCs leads to a slight upregulation of *Xist* [23]. It was shown later that deletion of intron 1 does not overtly abrogate *Xist* repression in male and female undifferentiated ESCs but seems involved in *Xist* induction and in preferential inactivation of the mutated allele upon differentiation [24,25]. Although minimal, OCT4 and SOX2 also bind *Tsix* at its *DxPas34* minisatellite region, and *Xite*, a gene encoding another long non-coding RNA that acts as a *Tsix* enhancer (see Section 5.1) and is involved in *Tsix* upregulation and concomitant *Xist* downregulation [29]. Finally, in ESCs, OCT4, NANOG, and SOX2 also downregulate the expression of *Rnf12*, an important X-linked XCI activator ([30,31]; see Section 4.1).

Additional pluripotency factors have also been implicated in the regulation of XCI initiation. REX1 clearly binds to the *DxPas34* region of *Tsix* in female cells and to the *Xist* promoter and its promoter distal region (Figure 2A) [32,33]. *Rex1* KD leads to reduced RNA Pol II recruitment to *DxPas34* and the *Tsix* 3′ regions as well as reduced H3K36me3 deposition, but no reduction at the *Tsix* TSS, indicating that REX1 is involved in *Tsix* elongation [32]. In addition, luciferase studies of the *Xist* promoter in conjunction with REX1 overexpression also indicated the direct role of REX1 in *Xist* repression [33]. REX1 overexpression studies in female ESCs led to the conclusion that REX1 is an activator of *Tsix* and a repressor of *Xist*. Moreover, overexpressing REX1 in *Tsix*-Cherry *Xist*-GFP ESCs, where the regular relationship between *Tsix* and *Xist* is absent, also showed REX1’s role in regulating *Xist* and *Tsix* independently [34]. Indeed, *Rex1* KO cells exhibit an increased number of *Xist* clouds and double clouds at the ESC stage and during differentiation [35]. Nevertheless, *Rex1* KO mice are fertile and viable, pointing to the robustness of XCI when perturbed during embryonic development. Finally, the pluripotency factors KLF4 and C-MYC also bind the 5′ region of *Tsix* [32].

The transcriptional regulator PRDM14, expressed in ESCs and primordial germ cells, and involved in reprogramming, has been reported to regulate *Xist* expression [36,37,38]. PRDM14 binds *Xist* intron 1 DNA, while *Prdm14* KD leads to upregulation of *Xist* in female ESCs. However, its mechanism of action might be indirect, by repressing *Rnf12* in ESCs through decreased binding of PRC2 and concomitant decreased H3K27me3 deposition around the *Rnf12* promoter region. However, *Prdm14* KO was reported not to affect *Xist* expression in another study [38].

Another autosomally encoded TF repressor of *Xist* is CTCF, an 11-Zn finger protein involved in a wide variety of gene regulatory mechanisms, such as insulation, gene activation and repression, and in setting up and maintaining the higher-order chromatin architecture, such as the boundary formation of topologically associating domains (TADs) (reviewed in [39] and explained further in Section 6.3). In silico analysis of the *Tsix DxPas34* and *Xist* promoter regions showed that these regions contain many CTCF consensus binding sequences, which bind CTCF in vitro and in vivo (Figure 2A) [20,40,41]. Furthermore, while *Ctcf* KD leads to *Tsix* downregulation and *Xist* upregulation, its overexpression leads to *Xist* downregulation, all pointing to CTCF acting as a repressor of *Xist* [40]. Its mechanism of action seems to be counteracted by the lncRNA *Jpx*/*Enox* (see Section 5.2), an activator of *Xist*, which titrates CTCF down from the *Xist* locus, leading to *Xist* upregulation upon differentiation [41]. Finally, deletion of a strong CTCF binding site in a region called RS14 at the *Tsix*/*Xist* junction leads to the absence of XCI from the mutant X in heterozygous differentiating female ESCs [42].

Finally, the MSL complex regulates sex determination and dosage compensation in *Drosophila* and is composed of several subunits, among which are MOF, MSL1, and MSL2, the latter binding DNA directly (reviewed in [43]). Dosage compensation in *Drosophila* is achieved by the MSL complex being recruited to the single male X chromosome, depositing H4K16ac through the histone acetyltransferase activity of MOF and concomitant X-linked gene upregulation. Analysis of the binding patterns of MSL1/2 and MOF in mouse ESC cells showed their prominent recruitment to the *Tsix DxPas34* region [44]. *Msl2* KD results in decreased H4K16ac in this region, followed by decreased *Tsix* expression and increased *Xist* expression. The authors then show that MSL2 facilitates proper recruitment of REX1 to *DxPas34* and the general transcription factor YY1 (see next section) to *Tsix*’s promoter. MSL2 removal also leads to chaotic XCI, with an increase in female differentiating ESCs bearing two *Xist* clouds. The authors propose a mechanism where the MSL complex binds *Tsix*, enhancing its expression and thus repressing *Xist* indirectly. However, several other factors involved in direct or indirect regulation of *Xist* also seem to be misregulated in *Msl2* KD experiments, making the exact role of the MSL complex in *Xist* regulation somewhat complex.

Altogether, these findings suggest that a set of autosomally encoded transcription factors act in conjunction to keep *Xist* repressed through direct and indirect mechanisms. While the pluripotency network is intricately associated with the repression of *Xist* at the ESC stage, its disappearance upon differentiation releases *Xist*’s repression, permitting its upregulation and XCI.

### 3.3. Autosomally Encoded TFs: Xist Activators

One of the best-studied autosomally encoded activators of *Xist* expression is YY1, a general transcription factor involved in context-dependent gene activation and repression (reviewed in [45]). Interestingly, REX1 is a paralog of YY1, where REX1 arose through a retrotransposition event in mammals, and both proteins share a highly similar consensus binding motif [46]. This might also explain why YY1 binds the *Tsix DxPas34* region and downstream of the *Xist* TSS as REX1 does (Figure 2B) [20,32]. Making use of a *Tsix*-STOP construct that displays *Xist* upregulation from the single X chromosome in male cells, a study showed that YY1 binds downstream of the *Xist* TSS both before and during XCI [20]. In contrast, YY1 recruitment disappears in differentiated male control ESCs carrying a silenced *Xist* locus on the single Xa. YY1 deposition is DNA methylation-dependent. This suggests a role for YY1 as an activator of *Xist* on the Xi, which was confirmed with *Yy1* KD studies in differentiating female ESCs [20] and by another study [47]. In line with this, *Yy1* KD studies in female MEFs revealed that YY1 is important for *Xist* maintenance expression or localization [20,48]. This study described an elegant mechanism for the role of YY1 vs. REX1 in *Xist* expression, where both proteins compete for the same binding sequences around the *Xist* TSS, with YY1 acting as an activator and REX1 acting as a repressor. REX1 downregulation upon exit of pluripotency leads to increased YY1 binding, resulting in *Xist* upregulation.

As stated above, several autosomally encoded members of the GATA TF family (GATA2-6) have been shown to regulate *Xist* expression [27]. Upregulation of GATA members 1 to 6 by dCRISPR-VP64 leads to increased *Xist* expression in mouse ESCs, potentially explained by extensive cross-activation of the members. For instance, independent overexpression of all GATA members consistently results in upregulation of *Gata4* and *Gata6*. In an independent GATA6-inducible overexpression system, *Xist* was upregulated in a dose-dependent manner within a few hours. CUT&TAG experiments indicated that GATA 2, 3, 4, and 6 bind separate distal *Xist* enhancers (RE79 and RE97) in extraembryonic cells. Since mouse ESCs show negligible GATA TF expression, the authors overexpressed GATA6 and detected its binding to RE79 only. This study also shows that the removal of RE79 and RE97 abrogates *Xist* expression during iXCI in vivo, and given that they are bound by several of the GATA factors (GATA2 and 3 in trophectoderm; GATA4 and 6 in primitive endoderm), the latter might be important for Xist sustained expression in a wide variety of differentiated tissues where these factors are expressed. In conclusion, GATA TF family members are thus potent activators of *Xist* in iXCI in extraembryonic tissues and, when overexpressed, in mouse ESCs [27].

## 4. Non-DNA-Binding Factors That Impact *Xist* Upregulation

### 4.1. X-Linked Protein Regulators: XCI Activators

The stochastic model for X chromosome counting postulates the presence of X-linked XCI activators whose workings are counterbalanced by autosomally encoded repressors. Studies during the last couple of decades by two independent groups have shown that the E3 ubiquitin ligase RNF12/RLIM acts as an X-linked activator of XCI. Ectopic insertion of BACs harboring *Rnf12* in male and female ESCs revealed how RNF12 overexpression leads to dosage-dependent upregulation of *Xist* [30]. Female *Rnf12* heterozygous cells, phenotypically identical to male cells in terms of *Rnf12* expression, manage to inactivate an X chromosome, albeit at a reduced rate, indicating the presence of additional XCI activators [24]. However, contradicting data were provided as to whether *Rnf12* KO ESCs fail to inactivate an X chromosome in vitro [24,35,49]. Truncation of *Rnf12* by a Neo cassette or complete removal of the ORF of *Rnf12* results in failure to upregulate *Xist* in vitro [24,35], whereas another study shows that RNF12 is dispensable for XCI in the epiblast [49]. These discrepancies might be explained by the different backgrounds of the mice used or by differences in developmental and in vitro differentiation conditions, such as RA-induced differentiation, EB differentiation, or low O2 levels that may affect the robustness of XCI [50]. From these results, it is clear that in vivo XCI is much more robust than in vitro XCI. Both groups agree, however, that imprinted *Xist* expression is highly dependent on *Rnf12* expression in vivo [35,51]. The mechanism through which RNF12 is implicated in *Xist* expression was clarified by conducting immunoprecipitation followed by mass spectrometry. This analysis revealed that REX1 is the primary TF co-purifying with RNF12 in ESCs [33]. Subsequent experiments revealed that RNF12 ubiquitinates REX1, targeting it for proteasomal degradation, in line with the increased REX1 stabilization found in *Rnf12* KO ESCs [33,35]. Moreover, the pluripotency factors OCT4 and NANOG have also been shown to repress *Rnf12* in ESCs (Figure 2A) [24,31], leading to a slight upregulation of *Rnf12* upon differentiation in female cells. Altogether, these results have led to the proposal of the existence of an RNF12/REX1 axis that regulates *Xist* expression. Increased levels of RNF12 lead to decreased REX1 levels, which release *Xist* repression, resulting in increased *Xist* levels and the initiation of XCI. In fact, removal of both *Rnf12* and *Rex1* partially rescues the *Rnf12* phenotype [35], further suggesting the presence of additional X-encoded activators of XCI.

Indeed, two recent studies have implicated the role of two X-linked but XCI-escaping histone demethylases, KDM5C/JARID1C and KDM6A/UTX, implicated in sexual differentiation (see review [52]) with opposing roles in enhancing *Xist* expression [21,53]. While KDM5C is an H3K4 demethylase, KDM6A is an H3K27 demethylase [54,55]. *Kdmc5* KO mice show female-specific lethality, and E5.5 epiblast cells display fewer or smaller *Xist* RNA clouds, suggesting a defect in *Xist* RNA expression [21]. In vitro differentiation of *Kdm5c* KO ESCs to epiblast-like cells (EpiLCs) also results in reduced *Xist* upregulation, while ectopic KDMC5 expression in *Tsix* KO sensitized male cells leads to increased *Xist* expression. In addition, KDM5C is strongly recruited to the *Xist* P2 promoter, leading to H3K4me2/3 demethylation into H3K4me1, along with an increase in H3K27ac, which are common histone modifications of active enhancers (Figure 2B). The authors propose that the *Xist* P2 promoter acts as an enhancer-like region of *Xist*. Finally, *Kdm5c* KO female epiblasts and EpiLCs show residual *Xist* expression, indicating again that several mechanisms relying on different X-encoded dose-dependent XCI activators are at play.

A recent preprint shows how another escaping gene, *Kdm6a*, might be involved in *Xist* expression regulation. *Kdm6a* KO ESCs exhibit female-specific impaired differentiation in vitro, decreased *Xist* expression levels with a concomitant reduction in *Xist* clouds, and impaired silencing of X-linked genes [53,56]. The authors propose a model where a double dosage of KDM6A in ESCs leads to H3K27me3 removal from the *Xist* promoter region, allowing for its upregulation. However, there is little KDM6A recruitment to the *Xist* promoter at days 2 and 7 of differentiation, which is when *Xist* expression is already affected (Figure 2B). This result and how KDM6A affects other players of XCI in the Xic will need to be further addressed.

KDM5C and KDM6A thus seem to be XCI activators through different mechanisms, and their being X-linked is in accordance with the stochastic model of *Xist* activation, inducing persistent *Xist* expression in female cells upon differentiation. However, since both genes escape XCI, they cannot participate in the feedback loop where decreasing X-linked activator expression upon monoallelic inactivation prevents upregulation of the other *Xist* allele.

### 4.2. Autosomally Encoded Proteins: Xist Repressors and Activators

Groundbreaking work by several groups described the large multidomain SPEN/SHARP as a critical factor in *Xist*’s silencing of the X chromosome [57,58,59,60,61]. While SPEN interacts with several co-repressors, such as the NCoR2/SMRT complex and chromatin remodelers and deacetylases such as NURD [58,62], it interacts through its SPOC domain with *Xist*’s RNA A repeat region to activate HDAC3 on the X, leading to silencing of gene expression ([63]; reviewed in [64]). Interestingly, *Spen* KO female ESCs fail to upregulate *Xist* expression upon differentiation [65]. Because SPEN binds the *Tsix* promoter region (Figure 2B) [63], the authors postulated a mechanism whereby SPEN silences *Tsix*, allowing for *Xist* upregulation upon differentiation. This was also predicted in a previous systems biology analysis of XCI [66]. Indeed, *Spen* KO differentiating ESCs show increased *Tsix* expression compared to WT cells, and co-removal of *Tsix* and *Spen* leads to the rescue of *Xist* expression and the formation of *Xist* clouds [65]. In contrast, a subsequent study showed that rapid SPEN removal by AID-Auxin led to increased *Xist* expression [67]. The discrepancy in results might be due to the differentiation conditions, different approaches, and/or timing of SPEN removal.

Chromatin remodelers play crucial roles in modulating the compaction and relaxation of chromatin during differentiation and development, facilitating or impeding access of TFs to chromatin and their binding targets. Chromatin remodeler CHD8 binds to the *Xist* promoter region in undifferentiated ESCs, and its removal by KD or KO in the pluripotent state leads to *Xist* expression reduction due to reduced chromatin accessibility, suggesting a role for CHD8 in activating *Xist* [47]. However, its role in *Xist* expression during differentiation seems more complex since *Cdh8* KO cells show a reverse phenotype, i.e., increased *Xist* expression, seemingly due to increased chromatin accessibility and YY1 binding to the *Xist* promoter (Figure 2A,B). Which other factors see their recruitment to the *Xist* promoter region change upon CHD8 removal? is an interesting question that needs further research. All in all, CHD8 seems to fine-tune *Xist* expression in the pluripotency stage and during differentiation.

Another group of interesting factors involved in *Xist* expression are RIF1 and KAP1/TRIM28. RIF1 and TRIM28 are versatile proteins: RIF1 is involved in wide-ranging mechanisms, such as replication timing, gene expression, chromatin contacts, and G-quadruplexes [68,69,70], while KAP1/TRIM28 is an epigenetic co-repressor involved in genomic integrity, DNA repair, imprinting, and other processes (reviewed in [71]). Dynamic interplay and recruitment of these factors to the *Xist* P2 region tilts the balance towards *Xist* upregulation on one allele or the other. Dynamic RIF1 recruitment is asymmetrically broken by KAP1 binding at the P2 promoter of one allele, leading to higher *Tsix* steady-state levels in *cis* and further removal of RIF1 from that allele, preventing *Xist* from upregulating (Figure 2A,B) [72]. This does not seem to be *Xist* P2 H3K9me-mediated. On the other allele, however, because KAP1 does not bind to it, *Tsix* is not upregulated, leading to further stable binding of RIF1 and *Xist* upregulation. The authors describe data supporting this complex bookmarking model. However, the exact mechanism of KAP1 recruitment to the Xic and its modulation of *Tsix* levels are still open questions. The concurrent interplay between RIF1, KAP1, *Xist*, and *Tsix* leads to a break in the symmetry of *Tsix* and *Xist* expression and Xa/Xi choice.

## 5. Long Non-Coding RNAs That Impact *Xist* Expression

### 5.1. lncRNA Xist Repressors

**Figure 3:**
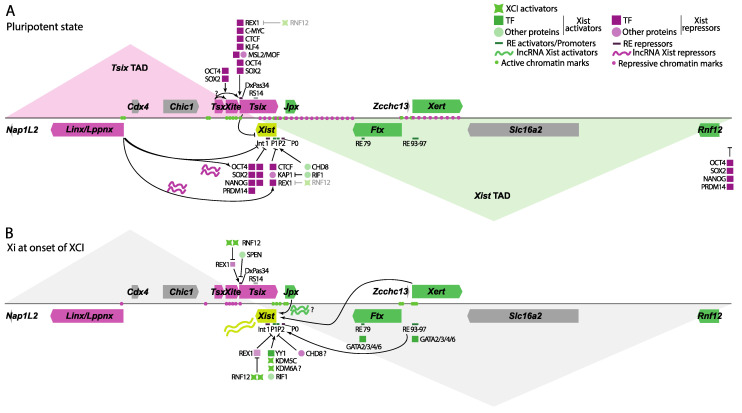
Overview of the different proteins, REs, lncRNAs, some local chromatin modifications, and topological organization of the Xic. Same legend as in Figure 2, plus the role of the different lncRNAs on *Xist* expression, the presence of different activating or repressing histone marks, and the bipartite TAD structure of the Xic. (**A**) In pluripotency, *Tsix* represses *Xist* thanks to transcription running through its gene body. *Tsx* seems to prop up *Xite* and *Tsix* expression, while *Linx*/*Lppnx* seem to have a role on *Xist* expression directly or through its RNA molecule at the level of OCT4 and REX1 displacement from the *Xist* locus. The Xic presents a bipartite TAD structure separated by a region at the 3′ end of *Xist* known as RS14. Expression of *Tsix* deposits the heterochromatic and euchromatic histone PTMs H3K36me3 and H3K4me2 across the *Tsix*/*Xist* gene body as well as the P1 and P2 promoters of *Xist*, maintaining the promoter regions in a poised state. Other histone heterochromatic PTMs found at the P1 and P2 promoters of *Xist* in pluripotency are H3K9me2, H3K9me3, H3K27me1, and H4K20me2. Repression of known *Xist* lncRNA activators is achieved by extended deposition of the heterochromatic mark H3K27me3 across the *Xist* TAD, while expression of known *Tsix* lncRNA activators is maintained by enrichment of the histone euchromatic marks H3K4me3 and H3K27ac. The *Tsix* TAD is depicted by the pink triangle, while the *Xist* TAD is depicted by the green triangle. When the lncRNA gene acts on *Tsix* or *Xist* through its lncRNA molecule, the lncRNA symbol has been added. (**B**) At the onset of XCI, in the future Xi, *Ftx* and *Xert* will help in *Xist* upregulation through either transcription running through their gene bodies (*Ftx*) or through RE at their promoters (*Xert*), while the mechanism of action of *Jpx* through its RNA molecule is debated. Loss of *Tsix* expression results in the enrichment of the histone euchromatic marks H3K4me2, H3K4me3, and H3K27ac at the *Xist* P1 and P2 promoters. *Tsix* downregulation is then maintained by the removal of the euchromatic mark H3K27ac and vague deposition of H3K27me3 at its promoter. Upregulation of known *Xist* lncRNA activators is achieved by depletion of the H3K27me3 hotspot within the *Xist* TAD and subsequent increase in the histone euchromatic PTMs H3K27ac and H3K4me3 at the lncRNA promoters. Subsequently, the downregulation of *Tsix* lncRNA activators is maintained by the deposition of H3K27me3. Finally, the bipartite TAD structure of the Xic is lost (grey). Shades of magenta show *Xist* repressors, while shades of green indicate *Xist* activators and XCI activators. When the lncRNA gene acts on *Tsix* or *Xist* through its lncRNA molecule, the lncRNA symbol has been added.

The Xic harbors several lncRNAs that act as modulators of *Xist* expression. These are spatially segregated into two TADs known as the *Tsix* TAD and the *Xist* TAD, harboring *Xist* activators and repressors, respectively (Figure 3, see Section 6.3). LncRNAs mostly regulate *Xist* in *cis*, either through transcription, enhancer-promoter and promoter-promoter interactions, or chromatin modifications. The most studied and known lncRNA that regulates *Xist* is *Tsix*, its antisense gene, with which it forms a binary switch. In ESCs, both genes are expressed biallelically, with high and low *Tsix* and *Xist* levels, respectively. However, at XCI onset, *Tsix* is downregulated and switched off from one of the alleles concurrently with *Xist* upregulation; this chromosome will become the Xi (Figure 1 and Figure 3B). Conversely, on the other chromosome, *Tsix* stays on, preventing upregulation of *Xist*; this chromosome will become the Xa. The role of *Tsix* in *Xist* expression was elucidated by means of targeted deletions of the *Tsix* promoter region or insertions to truncate its transcriptional unit, skewing that chromosome for inactivation by upregulation of *Xist* upon differentiation [14,73,74]. Interestingly, *Tsix* exerts its role on *Xist* not by its lncRNA molecule itself but by the act of transcription running through the *Xist* promoter [75]. Transcription of *Tsix* was later found to establish a transient repressive chromatin state on *Xist*, since *Tsix* truncation leads to increased H3K4me2/3 and H3K9ac deposition around the *Xist* promoter region, with decreased H3K9me, H3K27me3, and DNA methylation (see Section 6.1) [76,77,78,79,80]. Interestingly, introducing a polyadenylation signal in *Tsix* just 1 kb downstream of the *Xist* promoter leads to the same effect, indicating that it is antisense transcription running through the *Xist* promoter that establishes these repressive chromatin marks, impeding *Xist* upregulation [80]. On the other hand, forced upregulation of *Tsix* on one chromosome forces this X chromosome to stay active, while the WT chromosome is always inactivated [74]. The transcriptional interference mechanism was further confirmed and simulated in silico in a later study [4]. Finally, conformational switching between a more compact or relaxed *Tsix* TAD (see Section 6.3) results in breakage of the *Tsix*/*Xist* binary switch, with differential *Tsix* and *Xist* expression between the chromosomes [81]. A more compact TAD is associated with higher *Tsix* expression levels, probably due to more *cis* interactions with distal regulatory elements. It is now well established that *Tsix* acts as a repressor of *Xist* in *cis*.

Another lncRNA called *Xite*, located upstream of *Tsix*, acts as a *Tsix*-specific enhancer, regulating XCI choice [82]. Deletion of *Xite*, but not its truncation via a splice acceptor and polyadenylation signal, results in lowered *Tsix* expression in *cis* upon exit of pluripotency, forcing skewed XCI of the mutated chromosome, indicating that the promoter of *Xite* acts as an enhancer of *Tsix* (Figure 3A). It seems, however, that it does not regulate *Tsix* at the ESC stage, probably because *Tsix*’s expression is high and is involved in *Tsix* expression persistence upon differentiation. Moreover, as one would expect from an enhancer, its role in *Xist* repression is relatively smaller compared to *Tsix* since heterozygous deletions of the *Xite* promoter region skew XCI somewhat towards the X chromosome with the mutation, while *Tsix* mutants show full skewing.

Deletion of a 245 kb region upstream of *Tsix* results in decreased *Tsix* expression and increased *Xist* expression [83]. Also, deletion of that region in *Tsix*-Cherry *Xist*-GFP ESCs where the regular relationship between *Tsix* and *Xist* is absent also leads to *Xist* upregulation, suggesting the presence of a *Xist* repressor that is *Tsix* independent in the *Tsix* TAD. Subsequent analysis of the lncRNA *Linx* located within that region showed that the *Linx* promoter (*Linx*P), not its transcripts or transcription, represses *Xist* and thus the choice of which X is inactivated (Figure 3A) [83,84]. Given that no long-range contacts were detected between *Linx*P and the *Xist* promoter, how this is achieved mechanistically remains unclear. Interestingly, when a 2 kb construct of *Linx*P was inserted upstream of *Xist* at different distances, *Xist* expression was upregulated [83]. It seems thus that *Linx*P has a dual role as a silencer or enhancer, which is genomic context- or TAD-dependent. Another recent study found a similar, if not identical, lncRNA in the same region, *Lppnx*, whose promoter deletion led to increased *Xist* expression from that allele [85]. The authors link this lncRNA to the X controlling element, Xce [86], a region upstream of *Xite* and *Tsix*. Depending on the background origin of the X chromosomes in female mice, XCI is not fully random. Some alleles are preferentially inactivated with respect to other alleles. The authors show that OCT4 is required for *Lppnx* expression and that deletion of *Xist* intron 1 results in rescue of the phenotype, suggesting that *Lppnx* acts through *Xist*’s intron 1. Indeed, deletion of *Lppnx* leads to decreased recruitment of OCT4 and REX1 to *Xist*’s intron 1 and *Tsix*’s *DxPas34*. Since the promoters of *Linx* and *Lppnx* are very close and multiple alternative splice transcripts are generated from that location, it is unclear whether these two transcripts involved in *Xist* repression and/or Xce are the same or different lncRNAs [87,88]. These results warrant additional research.

Finally, another known gene located between *Xite* and the protein coding gene *Chic1* is *Tsx*, a lncRNA expressed in many tissues; however, it seems to code for the TSX protein in testis (Figure 3A) [89]. Its deletion in ESCs leads to a mild increase in female and male cells with double and single *Xist* clouds, respectively, while also showing lower *Tsix* levels in female ESCs and upon differentiation. However, not much else is known about its mechanism of action.

To conclude, several lncRNAs present in the *Tsix* TAD act in unison to repress *Xist* directly or via *Tsix*. Asymmetries born from their expression and/or interactions with *Tsix* and *Xist* lead to *Xist* upregulation or skewing of XCI upon differentiation of ESCs.

### 5.2. lncRNA Xist Activators

Several other lncRNAs lie upstream of *Xist*, contained within the *Xist* TAD. One of the first ones from this set to be involved in *Xist* expression is *Jpx/Enox*, a lncRNA that lies ~10 kb upstream of *Xist* (Figure 3B). *Jpx*’s expression increases 10–20-fold in both male and female differentiating ESCs, following a similar pattern of expression as *Xist* and also escaping XCI [90,91]. RNA FISH experiments indicated that when expressed from the Xi, it follows a similar nuclear localization pattern as genes escaping XCI, with its nascent RNA detected adjacent to *Xist* accumulations or clouds. Heterozygous deletions of *Jpx*, however, seem to decrease *Jpx* expression from the WT allele, indicating a feedback mechanism in trans, while *Xist* cloud formation was very much abrogated compared to WT cells, suggesting a role for *Jpx* in activating *Xist* in trans too during differentiation. Knockdown of *Jpx* at the posttranscriptional level also led to reduced *Xist* expression, indicating an RNA-mediated mechanism of action and not through the act of transcription or as an enhancer [91]. Moreover, random transgene insertion of a *Jpx*-*Xist* construct in female cells resulted in an increased number of cells with 2–3 clouds, suggesting again a trans mechanism [92]. However, a different study cast doubt on its mechanism working solely in trans [93]. Indeed, a heterozygous deletion of a large piece of DNA upstream of *Xist* from *Jpx* to *Rnf12* does not compromise *Xist* upregulation from the WT chromosome, although its levels are reduced. This reduction of *Xist* expression in the *Jpx*-*Rnf12* heterozygous deletion is not rescued by introducing a *Jpx* transgene, suggesting *Jpx*’s effect might be in cis and not in trans. On the other hand, another study revisiting these results concluded that heterozygous deletion of the Xic spanning from *Tsx* to *Ftx* (see next paragraph), and thus including *Jpx*, abrogates XCI [94]. Finally, another group recently reported that *Xist* expression in post-XCI cells is *Jpx*-dependent, but nascent *Xist* transcription is unmodified, suggesting that *Jpx* acts on *Xist* at the posttranscriptional level [95]. Why such results contradict each other remains an open question, though these differences might be due to the different background strains for each chromosome, the parental origin of the mutated chromosomes, and/or the differentiation conditions. They concur that *Jpx* acts as an activator of *Xist*, but its exact mechanism of action is still debated.

The discovery of another gene upstream of *Xist*, called *Ftx*, with no ORF and whose RNA is predominantly nuclear, suggested it might be a lncRNA [96]. Its expression increases during the differentiation of female ESCs, mirroring *Xist*, while staying mostly unchanged in differentiating male cells. As *Jpx*, it partially escapes XCI. *Ftx* removal by knocking out its first five exons and alternative promoters in male cells results in decreased H3K4me2 deposition and increased CpG methylation around the *Xist* promoter, both of which result in decreased *Xist* expression [96]. *Ftx* heterozygous and homozygous deletions in female cells lead to impaired *Xist* upregulation [97]. Analysis of the 3D conformation of the region shows strong interactions between the *Ftx* and *Xist* promoters in female undifferentiated ESCs. Directing a dCAS9 fused to KRAB to the *Ftx* promoter also results in *Xist* downregulation, indicating that *Ftx* transcription or its lncRNA is important in cis for *Xist* expression (Figure 3B). Similar results were obtained in vivo in a separate study [98]. Deletion of *Ftx* led to compromised XCI in vivo, with many genes being expressed from the Xi, probably due to diminished *Xist* expression in many tissues analyzed. It thus seems that promoter-promoter interactions between the *Ftx* promoter and the *Xist* promoter in cis drive *Xist* expression in ESCs and differentiation, in vitro and in vivo [97,98].

As stated above (see Section 2.2), several regulatory elements around 75–150 kb upstream of the *Xist* TSS have recently been found to positively affect *Xist* expression [26]. Several relatively short lncRNAs are expressed from that region, newly named *Xert* (Figure 3B). The main TSS is enriched with typical enhancer chromatin marks, such as H3K27ac and H3K4me1. *Xert* is upregulated along with *Xist*, *Jpx*, and *Ftx* at the onset of differentiation. This expression profile suggests co-regulation of *Xist* as *Jpx* and *Ftx* in cis. Transient silencing of *Xert* using CRISPRi indeed leads to decreased *Xist* expression in differentiating female cells, while its overexpression in male cells results in increased *Xist* expression. By deleting the *Xert* promoter region, the authors conclude that its effect on *Xist* expression is in cis [26]. In addition, increased interactions between *Xist* and *Xert* were reported during differentiation, further supporting its role as a *Xist* enhancer. Although these findings suggest that *Xert* exerts its effect on *Xist* upregulation through its enhancer capabilities, a potential role in *Xist* regulation for the *Xert* RNA or the act of transcription through the locus remains to be studied.

It is thus clear that several mechanisms are at play that regulate *Xist* and *Tsix* expression during the onset of XCI, such as the lncRNA product, the promoter of the lncRNA acting as an enhancer, or transcription running through the locus. These studies exemplify how difficult it is to identify common principles of action among these players. To overcome this challenge, conducting ectopic expression experiments for these lncRNAs, knocking them down, introducing termination signals, etc. are necessary to elucidate their specific roles in *Xist* regulation.

## 6. The Chromatin Landscape at the Onset of Random XCI

### 6.1. Chromatin Landscape at the Tsix/Xist Tandem

As previously discussed, the regulation of *Xist* expression involves multiple factors that orchestrate changes in the chromatin landscape within the Xic. It is now evident that several histone post-transcriptional modifications (PTMs) contribute to the maintenance of symmetric *Tsix* expression while repressing *Xist* expression in the pluripotent state. Upon exit of pluripotency, localized changes in these PTMs within the future Xi assist in the downregulation of *Tsix* and concomitant upregulation of *Xist,* ultimately breaking symmetric *Tsix* expression.

The clearest evidence for the role of histone PTMs in maintaining asymmetric expression came from ChIP studies of the chromatin landscape at *Tsix* and *Xist*. Early studies in female trophoblast stem cells (TSCs), which express *Xist* from the paternal allele, revealed an enrichment of H3K4me2 at the P1 promoter of *Xist* on the paternal allele. Concomitantly, H3K4me2 is absent at the P1 promoter on the maternal allele [77]. In line with this, a reversed pattern was observed at the *Tsix* TSS, whereby H3K4me2 is enriched at the TSS of *Tsix* on the maternal allele while absent from the paternal allele. The same study then explored the role of *Tsix* in the establishment of H3K4me2. Male mouse ESCs carrying a stop signal near the TSS of *Tsix*, thus truncating its expression, show a gain of H3K4me2 at the *Xist* P1 promoter. Moreover, a reduction of H3K4me2 across the *Tsix*/*Xist* tandem was also observed, highlighting the role of *Tsix* in modifying the histone PTM landscape at the tandem (Figure 3A,B). Similar results were shown in a separate study that same year in male MEFs, which do not express *Xist*, where the introduction of a stop signal near the TSS of *Tsix* leads to a gain in H3K4me2 at both the P1 and P2 promoters of *Xist* [79].

Follow-up investigations into *Tsix*’s mechanism for modifying the chromatin landscape elucidated that it is the transcriptional activity of *Tsix* across the *Xist*’s promoters rather than its lncRNA product that instigates these modifications. In accordance with previous findings and as stated in Section 5.1, the insertion of a stop signal near the *Xist* P2 promoter in male mouse ESCs, thus allowing for 93% transcription of *Tsix*, shows a gain in H3K4me2 at both the P1 and P2 promoters of *Xist* [80]. Moreover, a loss in H3K9me2 at both *Xist* promoters was also observed in the transgenic male lines alongside diminished DNA methylation, pointing towards the maintenance of a complex balance between euchromatic and heterochromatic PTMs by the transcription of *Tsix* across *Xist*’s promoter regions in the pluripotent state (Figure 3A).

Further exploration of histone PTMs induced by *Tsix* expression later revealed a role for H3K36me3 and H3K27me3 in repressing *Xist* expression. H3K36me3 is enriched across *Tsix* and highly enriched over the *Xist* promoter in male mouse ESCs in the pluripotent state. When *Tsix* is genetically deleted, enrichment of H3K36me3 is lost at the *Xist* promoters [99]. Interestingly, the same study finds enrichment of H3K27me3 at the *Xist* promoters in the transgenic lines, perhaps due to the expansion of the H3K27me3 hotspot upstream of *Xist* (see next section), indicating that *Tsix* transcription may have a bivalent role at the *Xist* promoters in maintaining both a euchromatic state by preventing H3K27me3 spread and a heterochromatic state by depositing H3K36me3 (Figure 3A). It is now known that H3K27me3 plays a critical role during iXCI. Early during oogenesis, an H3K27me3 domain is established, spanning *Xist* and upstream sequences up to *Rnf12.* This mark is maintained throughout the first cell divisions after fertilization, preventing the upregulation of *Xist* from the maternal X chromosome [100]. Indeed, its loss results in aberrant maternal XCI in pre-implantation development. The imprint that keeps the maternally inherited *Xist* allele inactive during mouse iXCI is thus polycomb-dependent H3K27me3 deposition at *Xist* and its upstream sequences. Finally, a comprehensive ChIP-seq analysis of histone PTM induced by *Tsix* has confirmed the complex interplay between euchromatic and heterochromatic marks governing the *Xist* promoter. Ectopic *Tsix* upregulation with a doxycycline-inducible system in EpiSCs, which have already initiated XCI, leads to diminished enrichment of H3K4me2 and increased enrichment of H3K36me3 at both promoters of *Xist* [101], confirming previous results. Additionally, this study further expanded on the changes in histone PTMs at the *Xist* promoters by *Tsix*. At endogenous *Xist* expression, the euchromatic marks H3K4me3 and H3K27ac are particularly enriched at the P2 promoter of *Xist*. Concomitantly, *Tsix* induction by doxycycline diminishes the presence of the euchromatic marks, resulting in the enrichment of the heterochromatic marks H3K9me2, H3K9me3, H3K27me1, and H4K20me2. It is worth noting that other dynamic chromatic changes may also play a role in the regulation of *Tsix*. Despite not being the primary focus of these studies, both forced *Xist* upregulation and exit of pluripotency show a slight gain of H3K27me3 and diminished H3K27ac at the *Tsix* TSS, most likely via SPEN [26,65,102], indicating a transition towards a heterochromatic state (Figure 3B).

An important mechanism in the epigenetic control of promoters is DNA methylation. The *Xist* promoter in ESCs is decorated partially with DNA methylation [10]. As with histone marks, *Tsix* blocks euchromatinization of the *Xist* promoter in ESCs by triggering DNA methylation in the ESC state. Indeed, the removal of *Tsix* in male ESCs and in vivo leads to decreased or loss of CpG methylation at the *Xist* promoter region [78,79,80]. Upon differentiation and XCI, *Tsix* triggers DNA methylation at the *Xist* promoter on the future Xa, mediated by the de novo methyltransferase DNTM3A, maintaining it silenced [76]. As stated earlier, *Ftx* also plays a role in CpG methylation at the *Xist* promoter, since its deletion results in increased methylation in the ESC state [96]. In iXCI, however, the maternal *Xist* allele is kept silent in a DNA-methylation-independent mechanism, since deletion of *Dnmt3a/b* does not result in Xist or *Tsix* misexpression in extraembryonic tissues [103].

Taken together, these studies suggest both *Xist* P1 and P2 promoters are decorated with both heterochromatic and euchromatic PTMs, maintaining them in a poised state. At the onset of XCI, the removal of heterochromatic PTMs at these promoters occurs on the future Xi, aiding in the upregulation of *Xist*. On the other hand, the TSS of *Tsix* is decorated with euchromatic PTMs in the pluripotent state, aiding in symmetric *Tsix* expression from both X chromosomes. The onset of XCI then leads to the removal of euchromatic PTMs and later the deposition of heterochromatic PTMs at the *Tsix* TSS, resulting in *Tsix* downregulation in the future Xi.

### 6.2. Chromatin Landscape at Xist Regulators

Although studied to a lesser degree, alterations in histone PTMs outside the *Tsix*/*Xist* tandem within the Xic locus may also contribute to the regulation of *Xist* expression. Early ChIP studies identified an H3K27me3/H3K9me3 hotspot extending ~350 kb upstream of *Xist* in female mouse ESCs in the pluripotent state [104]. Initially, this discovery was intriguing since heterochromatic marks were conventionally associated with the Xi. The characterization of this H3K27me3 hotspot was further elucidated by follow-up studies in female mouse ESCs with skewed XCI upon exit of pluripotency. ChIP analysis of H3K27me3 female mouse ESCs showed enrichment of H3K27me3 from the 5′ end of the *Xist* gene up to the 5′ end of *Ftx* in the pluripotent state, further confirming the previous findings (Figure 3A) [105]. More importantly, the appearance and disappearance of the H3K27me3 hotspot correlate with pluripotency status, whereby exit of pluripotency shows ablation of the H3K27me3 hotspot, hinting at its plausible role in silencing *Xist* cis activators within the Xic in the pluripotent state.

Upon exit of pluripotency, loss of H3K27me3 at *Xist* cis activators within the Xic is accompanied by gain of euchromatic marks. Notably, *Jpx*, *Ftx*, and *Xert* (see Section 5.2) show increased H3K27ac and H3K4me3 at their TSSs [26,105]. Moreover, the RE93-97 enhancer region also gains H3K27ac and H3K4me1 (see Section 2.2), whereas another enhancer region, RE79, characterized by H3K27ac enrichment in TSCs, does not show H3K27ac accumulation during rXCI (Figure 3B) [27]. On the other hand, *Tsix* cis activators within the Xic exhibit a mirrored pattern, undergoing a loss of euchromatic and gain of heterochromatic marks upon exit of pluripotency. In the pluripotent state, the euchromatic marks H3K27ac and H3K4me3 are enriched at the TSS of *Linx* and across *Xite*, while *Tsx* does not show enrichment of these marks at its TSS but rather at an upstream region (Figure 3A) [26,83,102]. Exit of pluripotency shows depletion of these euchromatic marks on the Xi and concomitant gain of H3K27me3, most likely indicating their downregulation upon XCI kickoff (Figure 3B).

Taken together, these studies suggest that *Xist* cis activators within the Xic are decorated with a heterochromatic H3K27me hotspot in the pluripotent state, which is removed upon onset of XCI, while *Tsix* cis activators within the Xic show an inverse trend with respect to their chromatin state. Allele-specific re-analysis of publicly available ChIP-seq data in female mouse ESCs both during and upon exit of pluripotency can help us further elucidate the chromatin changes of Xic regulators upon onset of XCI.

### 6.3. Higher Chromatin Structure of the Xic

Advancements in chromosome conformation capture technologies have enabled the comprehensive assessment of chromosomal interactions, both locally and on a genome-wide scale (reviewed in [106]). Early insights on the higher chromatin organization of the Xic came from a 3C study conducted in mouse ESCs. This study demonstrated that *Xist* establishes contacts with the *Jpx* locus in the future Xi, while *Tsix* establishes contacts with the *Xite* locus in the future Xa upon exit of pluripotency [107]. It was then proposed that both *Xist* and *Tsix* segregate into two distinct regulatory hubs, facilitating the observed asymmetric switch of these lncRNAs during XCI.

This initial notion of a higher-order chromatin separation between *Xist* and *Tsix* was later confirmed by 5C and high-resolution microscopy studies [84]. We now know that both *Xist* and *Tsix* form two distinct TADs, facilitating their spatial segregation (Figure 3A). TADs are typically ~1 Mb in size and contain loop domains corresponding to recurrent or stable contacts between loci 5 to 500 kb apart [106]. The *Xist* TAD is ~550 kb in size and spans from the 3′ end of *Xist* to a region upstream of *Rnf12*, while the *Tsix* TAD is ~300 kb in size and spans from a region downstream of *Nap1L2* to the 3′ end of *Xist*. These TADs are separated by RS14 (see Section 3.2), which binds CTCF and forms the TAD boundary [42,84]. Gene expression within each TAD is highly correlated in female mouse ESCs throughout differentiation, indicating that this physical clustering coordinates gene expression patterns throughout development. Deletion of a 58 kb region containing the TAD boundary results in uncoordinated gene expression within each TAD and increased ectopic contacts between them. Interestingly, this deletion does not result in the merging of these two neighboring domains but rather seems to attenuate them, indicating that other elements contribute to defining them [84].

The relevance of the bipartite TAD organization of the Xic in the transition from symmetric to asymmetric gene expression of *Xist* and *Tsix* upon onset of XCI was later revealed from subsequent studies in both male and female mouse ESCs. Taking advantage of CRISPR-Cas9, a ~40 kb region was inverted within the Xic spanning from *Xist* P1 to the promoter of *Tsix*, thereby relocating both *Xist* and *Tsix* alongside their respective promoters into the opposite TAD [108]. This inversion does not seem to attenuate either’s TAD structure or alter their boundary. However, while transgenic lines do not exhibit altered *Tsix* expression in the pluripotent state, both male and female transgenic lines exhibit ectopic *Xist* upregulation. Moreover, prolonged *Tsix* expression throughout differentiation was observed in female transgenic lines. Consequently, these findings collectively suggest that inversion of the *Tsix*/*Xist* locus results in a *Xist* regulatory pattern resembling that of *Tsix* and vice versa, partly overriding the previously discussed *Xist* regulation rules such as inhibition by pluripotency and double gene dosage. This highlights the impact of the cis-regulatory landscape on the proper timing of gene expression patterns.

A recent transgenic study has evaluated the importance of the *Tsix* TAD topology on *Xist* regulation [109]. A 245 kb region was inverted, spanning from the downstream region of *Nap1L2* to the downstream region of *Tsx* in both male and female mouse ESCs, thus preserving the integrity of the *Xite* and *Tsix* loci. Comprehensive 5C analysis of the altered Xic points towards the formation of new but similar loop interactions within the *Tsix* TAD, altering its topology. Intriguingly, the new *Tsix* TAD topology leads to a gain of insulation across the *Tsix/Xist* TAD boundary. Inverted lines show *Tsix* downregulation in the pluripotent state, highlighting the relevance of the TAD topology on maintaining *Tsix* expression, although this effect was lost upon exit of pluripotency. Interestingly, both male and female inverted lines display ectopic *Xist* upregulation upon exit of pluripotency, but not in the pluripotent state, suggesting that changes in one TAD structure can influence the expression patterns of a neighboring TAD, proposing the existence of inter-TAD communication. However, it is likely that *Xist*’s observed misregulation upon exit of pluripotency is tightly linked to the downregulation of *Tsix* or other factors from the pluripotent state.

Taken together, these studies underscore the role of the higher chromatin organization within the Xic at the onset of XCI. TADs ensure correct temporal gene expression patterns both within and between neighboring TADs upon exit of pluripotency, guaranteeing *Xist* upregulation at the appropriate developmental stage.

## 7. Concluding Remarks

How female cells manage to count the number of X chromosomes per diploid genome and initiate inactivation has puzzled scientists for decades. Increasing amounts of data now support a stochastic model that breaks symmetric *Tsix*/*Xist* expression from a single X chromosome in female murine ESCs upon differentiation. Over the last decades, research has disentangled the role of autosomally encoded factors that repress or activate *Xist* expression and the role of X-linked XCI activators. A tightly regulated balance of trans and cis factors thus ensures a counting mechanism whereby a single X chromosome in diploid female cells upregulates *Xist* monoallelically, leading to a cascade of events that will eventually silence most genes along that single X. This then leads to the establishment of a negative feedback loop whereby the X-linked XCI activators are silenced on the Xi in cis, preventing inactivation of the remaining active X chromosome in *trans*. Several factors contribute to this cascade of events to kick off *Xist* upregulation monoallelically, and thus XCI. We believe these mechanisms work at different hierarchical levels: DNA elements, transcription factors, other regulatory proteins, lncRNAs, and the chromatin and topological landscape. See Supplementary Table S1 for a summary of all factors involved in *Xist* expression in this review. Disentangling the hierarchical relevance of each element is difficult, as they may cooperate synergistically or redundantly, but also in a timely manner.

Since cells in the pluripotent ESC state have two active X chromosomes, where *Xist* expression is kept biallelically low or repressed, the connection between the pluripotency factor network and *Xist* repression was self-evident. Evidence now indicates that the pluripotency factor network and other transcription factors and proteins aid in maintaining biallelic *Tsix* expression and thus indirectly inhibiting *Xist* expression in ESCs, while also directly inhibiting *Xist*. Downregulation of the pluripotency factor network seems to be one of the first cues for XCI kickoff. It is thus essential for the cell to integrate the pluripotency factor network, differentiation cues, and X dosage to establish the correct *Xist* expression pattern. DNA elements would consequently sense these cues in the form of protein factors binding to them, allowing for subsequent changes in the chromatin landscape and transcriptional outcomes, thus facilitating and locking in an asymmetric *Tsix*/*Xist* expression pattern upon differentiation. Moreover, although *Tsix* has a very distinct role in *Xist* inhibition, Xic lncRNAs generally seem to have a more modulating function in symmetry breaking between *Xist* and *Tsix*, with variability in their mechanism of action, be it the lncRNA molecule self, transcription, or promoter and enhancer sharing. The local topology of the Xic indicates its importance in the interplay between these different levels. Finally, further levels of *Xist* post-transcriptional control that fall outside the scope of this review are alternative splicing and *Xist* RNA molecular stability. Alternative splicing of *Xist* leads to a long and short isoform, both functional in XCI during the differentiation of ESCs [13], while *Xist* RNA stability modulation plays an important role in the downstream cascade of XCI [17,110].

While this review focuses on mouse rXCI, it is worth noting that differences have been described between mechanisms regulating XCI in different species. For example, while *Tsix* has a clear role in murine rXCI, this is not the case in humans, possibly due to the fact that the *TSIX* gene body does not fully overlap with that of *XIST,* and thus its transcription does not run through the *XIST* promoter. Moreover, female human cells in pre-implantation embryos display two clouds of *XIST* in vivo, while in vitro-grown human pluripotent stem cells display one or two *XIST* clouds depending on derivation procedures and culture conditions (reviewed in [1]). However, as with other mammals, human differentiated cells show a single inactive X chromosome with a single *XIST* cloud. Additionally, it has been shown that human *FTX* does not seem to play a role in *XIST* regulation, while *JPX* does regulate *XIST* through the act of transcription, possibly involving promoter or enhancer co-activation, and does not involve lncRNA-mediated regulation of *XIST* as described for mice [95]. Altogether, this highlights the similarities and differences between mouse and human XCI. It is also worth noting how, even within rodents, patterns of XCI and *Xist* expression may differ between species [111], illustrating the variety of mechanisms that lead to dosage compensation in mammals.

While more than three decades have passed since its discovery, understanding how *Xist* is regulated at the different levels of gene regulation explained in this review is still an ongoing task. Current open questions that warrant further research remain, such as the actual role of *Jpx* in *Xist* expression modulation and the differences and similarities between *Linx* and *Lppnx*. Are there also additional *Xist* and XCI activators? More of them will probably come to light in the future. It is also interesting to hypothesize whether any of the lncRNAs described in this review contain small ORFs that might be functional [112]. In vivo studies seem to point to *Xist* regulation as a highly redundant process. In other words, while in vitro deletions of cis elements or trans regulators misregulate *Xist* expression in mouse ESCs, in vivo deletions of several of these factors seem primarily redundant, suggesting the presence of very robust overlapping mechanisms. Given how essential XCI is for cell survival during female development, the presence of several redundant, “fool proof” mechanisms seems evolutionarily sensible.

## Data Availability

Data are contained within the article.

## Supplementary Materials

The following supporting information can be downloaded at: https://www.mdpi.com/article/10.3390/epigenomes8010006/s1. Table S1: Overview of the different factors involved in *Xist* expression control in mouse ESCs in rXCI.
