# Supplementary material for: Orchestrating Asymmetric Expression: Mechanisms behind Xist Regulation
Source: Epigenomes. 2024 Feb 1;8(1):6. doi: 10.3390/epigenomes8010006 (PMC10885031; doi:10.3390/epigenomes8010006)
Supplement: Supplementary file 1 [file epigenomes-08-00006-s001.zip › epigenomes-2809751-supplementary.pdf]

epigenomes

Review

Orchestrating Asymmetric Expression: Mechanisms behind *Xist* Regulation

Samuel Jesus Luchsinger-Morcelle, Joost Gribnau and Hegias Mira-Bontenbal *

Department of Developmental Biology, Erasmus MC, University Medical Center, 3015 GD Rotterdam, The Netherlands; s.luchsingermorcelle@erasmusmc.nl (S.J.L.-M.); j.gribnau@erasmusmc.nl (J.G.)
* Correspondence: h.mirabontenbal@erasmusmc.nl

Supplementary Table S1. Overview of the different factors involved in *Xist* expression control in mouse ESCs in rXCI.

Factor	Category	Coding	Regulation on *Xist*	Mode of action on *Xist*
P1	DNA element	X linked	Upregulation	Directly
P2	DNA element	X linked	Upregulation	Directly
P0	DNA element	X linked	Upregulation	Directly–alternative unstable Xist transcripts
Xist Intron 1	DNA element	X linked	Downregulation	Directly
RE79	DNA element	X linked	Upregulation	Directly
RE93-97	DNA element	X linked	Upregulation	Directly<br>Indirectly via *Xert* upregulation
OCT4	Transcription Factor	Autosomal	Downregulation	Directly via Xist intron 1<br>Indirectly via *Tsix* and *Xite* upregulation, RNF12 downregulation
SOX2	Transcription Factor	Autosomal	Downregulation	Directly via *Xist* intron 1<br>Indirectly via *Tsix* and *Xite* upregulation, RNF12 downregulation
NANOG	Transcription Factor	Autosomal	Downregulation	Directly via *Xist* intron 1<br>Indirectly via *Tsix* upregulation, *Rnf12* downregulation
REX1	Transcription Factor	Autosomal	Downregulation	Directly<br>Indirectly via *Tsix* upregulation
KLF4	Transcription Factor	Autosomal	Downregulation	Indirectly via *Tsix* upregulation
C-MYC	Transcription Factor	Autosomal	Downregulation	Indirectly via *Tsix* upregulation

PRDM14	Transcription Factor	Autosomal	Downregulation	Directly via *Xist* intron 1? Indirectly via *Rnf12* downregulation?
CTCF	Transcription Factor	Autosomal	Downregulation	Directly<br>Indirectly via *Tsix* upregulation
YY1	Transcription Factor	Autosomal	Upregulation	Directly<br>Indirectly via *Tsix* downregulation
GATA2/3/4/6	Transcription Factor	Autosomal	Upregulation	Indirectly via RE79/93-97
CHD8	Chromatin remodeller	Autosomal	Complex	Directly via accessibility modification of *Xist* promoter
KAP1	Scaffold protein	Autosomal	Downregulation	Indirectly via competition with RIF1 and *Tsix* stabilisation
RIF1	Multifaceted protein	Autosomal	Upregulation	Directly
MSL2/MOF	Histone acetyltransferase complex	Autosomal	Downregulation	Indirectly via H4K16ac deposition at *DxPas34*, YY1 recruitment to Tsix promoter and REX1 to *DxPas34*
SPEN	Chromatin remodeller	Autosomal	Upregulation	Indirectly via *Tsix* downregulation
RNF12	E3 ubiquitin ligase	X-Linked	Upregulation	Indirectly via REX1 degradation
GATA1	Transcription Factor	X-Linked	Upregulation	Indirectly via *Gata6* upregulation?
KDM5C	H3K4 demethylase	X-Linked	Upregulation	Directly via *Xist* promoter H3K4 demethylation
KDM6A	H3K27 demethylase	X-Linked	Upregulation	Directly via *Xist* promoter H3K27 demethylation?
Tsix	LncRNA	X-Linked	Downregulation	Direct via transcription through *Xist* promoter
Xite	LncRNA	X-Linked	Downregulation	Indirectly via *Tsix* upregulation–enhancer-like function
Tsx	LncRNA	X-Linked	Downregulation	Indirectly via *Tsix* upregulation?

Linx/Lppnx	LncRNA	X-Linked	Downregulation	Unclear/Direct *Xist* downregulation via intron 1-*DxPas34*/OCT4-REX1
Jpx	LncRNA	X-Linked	Upregulation	Unclear, RNA in *cis/trans*? at posttranscriptional level of *Xist?*
Ftx	LncRNA	X-Linked	Upregulation	Directly via promoter-promoter interactions, decreased DNA methylation at *Xist* promoter
Xert	LncRNA	X-Linked	Upregulation	Directly via enhancer-like function
H3K27me1	Histone PTM	N/A	Downregulation	Direct *Xist* downregualtion via *Xist* promoter heterochromatinisation
H3K27me3	Histone PTM	N/A	Downregulation<br><br><br><br><br><br>Upregulation	Direct *Xist* downregualtion via *Xist* promoter heterochromatinisation<br>Indirect *Xist* downregulation via *Jpx*, *Ftx*, and *Xert* heterechromatinisation<br>Indirect *Xist* upregulation via *Xite*, *Tsx*, and *Linx* heterechromatinisation
H4K20me2	Histone PTM	N/A	Downregulation	Directly via promoter heterochromatinisation
H3K9me2	Histone PTM	N/A	Downregulation	Directly via promoter heterochromatinisation
H3K9me3	Histone PTM	N/A	Downregulation	Directly via promoter heterochromatinisation
H3K36me3	Histone PTM	N/A	Downregulation	Directly via promoter heterochromatinisation
H3K4me2	Histone PTM	N/A	Upregulation	Directly via promoter euchromatinisation

H3K27ac	Histone PTM	N/A	Downregulation<br><br><br><br><br>Upregulation	Indirect Xist downregulation by *Tsix*, *Linx*, *Xite*, and *Tsx* heterochromatinization<br>Direct *Xist* upregulation via *Xist* promoter euchromatinisation<br>Indirect *Xist* upregulation via *Jpx*, *Ftx*, and *Xert* euchromatinisation
H3K4me3	Histone PTM	N/A	Downregulation<br><br><br><br><br>Upregulation	Indirect *Xist* downregulation by *Tsix*, *Linx*, *Xite*, and *Tsx* heterochromatization<br>Direct *Xist* upregulation via *Xist* promoter euchromatization<br>Indirect *Xist* upregulation via *Jpx*, *Ftx*, and *Xert* euchromatinisation
DNA methylation	DNA methylation	N/A	Downregulation	Directly
Tsix TAD	Higher chromatin structure	N/A	N/A	Ensuring *Tsix* expression in the pluripotent state
Xist TAD	Higher chromatin structure	N/A	N/A	Ensuring timely expression of *Xist* upon exit of pluripotency

Disclaimer/Publisher's Note: The statements, opinions and data contained in all publications are solely those of the individual author(s) and contributor(s) and not of MDPI and/or the editor(s). MDPI and/or the editor(s) disclaim responsibility for any injury to people or property resulting from any ideas, methods, instructions or products referred to in the content.
